# Genetic etiology of truncus arteriosus excluding 22q11.2 deletion syndrome and identification of c.1617del, a prevalent variant in *TMEM260*, in the Japanese population

**DOI:** 10.1038/s10038-024-01223-y

**Published:** 2024-02-13

**Authors:** Hisao Yaoita, Eiichiro Kawai, Jun Takayama, Shinya Iwasawa, Naoya Saijo, Masayuki Abiko, Kouta Suzuki, Masato Kimura, Akira Ozawa, Gen Tamiya, Shigeo Kure, Atsuo Kikuchi

**Affiliations:** 1 Department of Pediatrics, Tohoku University Graduate School of Medicine, Sendai, Japan; 2 Department of Pediatric Cardiology, Miyagi Children’s Hospital, Sendai, Japan; 3 Department of AI and Innovative Medicine, Tohoku University Graduate School of Medicine, Sendai, Japan; 4 Tohoku Medical Megabank organization, Tohoku University, Sendai, Japan; 5 Statistical Genetics Team, RIKEN Center for Advanced Intelligence Project, Tokyo, Japan; 6 Department of Rare Disease Genomics, Tohoku University Graduate School of Medicine, Sendai, Japan; 7 Department of Pediatrics, Yamagata University Graduate School of Medicine, Yamagata, Japan; 8 Miyagi Children’s Hospital, Sendai, Japan

**Keywords:** Congenital heart defects, Haplotypes, Clinical genetics, Next-generation sequencing, Medical genomics

## Abstract

Truncus Arteriosus (TA) is a congenital heart disease characterized by a single common blood vessel emerging from the right and left ventricles instead of the main pulmonary artery and aorta. TA accounts for 4% of all critical congenital heart diseases. The most common cause of TA is 22q11.2 deletion syndrome, accounting for 12–35% of all TA cases. However, no major causes of TA other than 22q11.2 deletion have been reported. We performed whole-genome sequencing of 11 Japanese patients having TA without 22q11.2 deletion. Among five patients, we identified pathogenic variants in *TMEM260*; the biallelic loss-of-function variants of which have recently been associated with structural heart defects and renal anomalies syndrome (SHDRA). In one patient, we identified a de novo pathogenic variant in *GATA6*, and in another patient, we identified a de novo probably pathogenic variant in *NOTCH1*. Notably, we identified a prevalent variant in *TMEM260* (ENST00000261556.6), c.1617del (p.Trp539Cysfs*9), in 8/22 alleles among the 11 patients. The c.1617del variant was estimated to occur approximately 23 kiloyears ago. Based on the allele frequency of the c.1617del variant in the Japanese population (0.36%), approximately 26% of Japanese patients afflicted with TA could harbor homozygous c.1617del variants. This study highlights *TMEM260*, especially c.1617del, as a major genetic cause of TA in the Japanese population.

## Introduction

Congenital heart disease (CHD) is one of the most common birth defects, affecting approximately 1% of newborns [1]. Its etiology is still unknown for the majority of cases. To date, genetic causes account for only 34% of all CHD cases, with aneuploidy, copy number variants (CNVs), and single-nucleotide variants (SNVs) accounting for 13%, 10%, and 10% of the cases, respectively [1].

Truncus Arteriosus (TA) is a CHD characterized by a single common blood vessel that emerges from the right and left ventricles instead of the main pulmonary artery and aorta. TA has an incidence of 3–10 per 100,000 live births and accounts for 4% of all critical CHDs [2]. The 22q11.2 deletion syndrome is a major cause of TA and is associated with 12% to 35% of TA cases [3]. *TBX1* is a gene responsible for CHDs of 22q11.2 deletion syndrome [4]. Apart from 22q11.2 deletion syndrome, several gene abnormalities, including *NKX2–5* [5], *NKX2–6* [6], and *GATA6* [7], have also been associated with TA. Recently, biallelic pathogenic variants of *TMEM260*, which encodes a multipass transmembrane protein, O-mannosyltransferase [8], have been associated with structural heart defects and renal anomalies syndrome (SHDRA syndrome; MIM #617478), including TA [8–12]. However, no major genetic causes of TA, other than 22q11.2 deletion, have been reported. The aim of this study was to explore the genetic causes of TA other than the 22q11.2 deletion in the Japanese population.

## Material and methods

### Patients

Eleven patients with unexplained TA were studied (Table 1). Patients with chromosome 22 abnormalities were excluded. This study was approved by the Ethics Committee of the Tohoku University School of Medicine and Tohoku Medical Megabank organization. Written informed consent was obtained from patients or their parents.

**Table 1:** Genetic analysis results and characteristics of 11 truncus arteriosus patients

Patient number	Patient 1	Patient 2	Patient 3	Patient 4	Patient 5	Patient 6	Patient 7	Patient 8	Patient 9	Patient 10	Patient 11
Race	Japanese	Japanese	Japanese	Japanese	Japanese	Japanese	Japanese	Japanese	Japanese	Japanese	Japanese
Gender	female	female	male	female	male	male	female	female	male	female	male
Age	3 months (deceased)	11 years	29 years	16 years	6 years	21 months	8 days	3 years	3 years	25 years	22 years
Gene	*TMEM260*	*TMEM260*	*TMEM260*	*TMEM260*	*TMEM260*	*GATA6*	*NOTCH1*	−	−	−	−
Accession number	ENST00000261556.6	ENST00000261556.6	ENST00000261556.6	ENST00000261556.6	ENST00000261556.6	ENST00000269216.3	ENST00000651671.1				
Genomic coordinates (GRCh37/hg19)	chr14:57099780TG>T	chr14:57099780TG>T	chr14:57099780TG>T	chr14:57099780TG>T	chr14:57099780TG>T	chr18:19761478G>A	chr9:139417499C>T	−	−	−	−
	chr14:57099780TG>T	chr14:57099780TG>T	chr14:57099780TG>T	chr14:57052611C>CT	chr14:57114051C>T						
cDNA coordinates	c.[1617del];[1617del]	c.[1617del];[1617del]	c.1617del(;)(1617del)	c.332dup(;)1617del	c.[1617del];[1960C>T]	c.[1367G>A];[1367=]	c.[545G>A];[545=]	−	−	−	−
Protein coordinates	p.[Trp539Cysfs*9];[Trp539Cysfs*9]	p.[Trp539Cysfs*9];[Trp539Cysfs*9]	p.Trp539Cysfs*9(;)Trp539Cysfs*9	p.(Thr112Hisfs*36)(;)Trp539Cysfs*9	p.[Trp539Cysfs*9];[(Gln654*)]	p.[(Arg456His)];[=]	p.[(Cys182Tyr)];[=]	−	−	−	−
Number of tested individuals	trio	trio	solo	solo	duo (mother)	trio	trio	trio	septet (mother, 5 brothers)	quad (parents, a sisiter)	solo
Inheritance	inherited	inherited	not provided	not provided	inherited	de novo	de novo	NA	NA	NA	NA
Family history of CHD	older sister : TA (deceased)	−	−	−	−	−	−	−	younger sister : DORV	−	−
Type of TA											
Collett and Edwards classification	Type II	Type I	Type I	Type II	Type I	Type I	Type III	Type I	Type II	−	−
Van Praagh classification	Type A2	Type A1	Type A1	Type A2	Type A1	Type A1	-	Type A1	Type A2	Type A3	Type A3
Combined cardiovascular malformation	Cor triatriatum	RAA	PAPVR	−	PAPVR	−	−	IAA (typeB)	−	MAPCA	MAPCA
	CAF				PLSVC						
	PAPVR										
	PLSVC										
Heart condition	post palliative Rastelli procedure	post intracardiac repair	post intracardiac repair	post intracardiac repair	post intracardiac repair	post intracardiac repair	post bilateral PA banding	post intracardiac repair	post intracardiac repair	post intracardiac repair	post intracardiac repair
Neurodevelopmental delay	NA	posthemorrhagic hydrocephalus	−	autism spectrum, ADHD	−	language developmental delay, dysplasia of cortical gyrus	NA	posthemorrhagic hydrocephalus	hyperactive tendency	−	−
Renal failure	post palliative Rastelli procedure	−	−	−	−	−	−	−	−	−	−
Oliguria	post palliative Rastelli procedure	−	−	−	−	−	−	−	−	−	−
Elevated creatinine level (normal:0.2−0.9 mg/dl)	−	−	−	−	−	−	−	−	NA	−	−
Proteinuria	−	−	−	−	−	NA	NA	−	−	NA	NA
Other features	−	enuresis, myopia, amblyopia, hearing impairment	atlantoaxial rotatory fixation	−	hydronephrosis	shortening of the lingual pedicel, left migrating testis, left polydactyly	−	−	−	chronic headache	hearing impairment

*CAF* Coronary artery fistula, *TA* Truncus arteriosus, *PAPVR* Partial anomalous pulmonary venous return, *PLSVC* Persistent left superior vena cava, *RAA* Right aortic arch, *PA* *banding* Pulmonary artery banding, *ADHD* Attention deficit hyperactivity disorder, *IAA* Interrupted aortic arch, *DORV* Double outlet right ventricle, *MAPCA*, Major aortopulmonary collateral artery, *NA* not applicable

### Genomic analysis

Genomic DNA was extracted from blood or saliva samples of patients and their families. We performed whole-genome sequencing (WGS) with DNBSEQ T7 (MGI Tech) in 150 bp paired-end mode using PCR-free libraries, according to previously described methods with minor modifications [13, 14]. The sequenced reads were mapped to the hg19 human reference genome using BWA MEM (ver 0.7.17-r1188). SNVs, short indels, and CNVs were called using the Genome Analysis Toolkit software v4.2.6.1, and structural variants were called using Smoove (v0.2.8). The variants were then annotated using SnpEff (v.5.1) after quality filtering. We considered introns ±100 bp from exons, CNVs, and structural variants. All candidate variants were confirmed by Sanger sequencing.

### Nonsense-mediated mRNA decay (NMD) inhibition

To test for NMD, Epstein-Barr virus transformed lymphoblastoid cell lines (EBV-LCLs) were established from Patient 1 and her parents using a previously described method [15]. The cells were treated with cycloheximide at a final concentration of 100 μg/ml. Cell lysates were harvested four hours after treatment. Total RNA was extracted from both treated and untreated cell lysates using the RNeasy Kit (Qiagen Inc., Valencia, CA, USA). RT-PCR was performed using a PrimeScript^TM^ RT Reagent Kit with gDNA eraser (Perfect Real Time) (TaKaRa, Shiga, Japan), followed by Sanger sequencing.

### Variant database surveys in various populations

The minor allele frequency of c.1617del in *TMEM260* in various populations, including Japanese, East Asian, and Southeast Asian populations, were obtained from public genome databases as follows: gnomAD v2.1.1 (https://gnomad.broadinstitute.org) for various populations [16], ToMMo 54KJPN (https://jmorp.megabank.tohoku.ac.jp) for the Japanese population [17], the ChinaMAP (http://www.mbiobank.com) for the Chinese population [18], Taiwan Biobank (https://taiwanview.twbiobank.org.tw) for the Taiwanese population [19], Korean Variant Archive 2 (KOVA 2, https://www.kobic.re.kr/kova) for the Korean population [20], Thai Reference Exome Database (T-REx, https://trex.nbt.or.th) for the Thai population [21], and the 1000 Vietnamese Genome Project (1KVG) (https://genome.vinbigdata.org/about) for the Vietnamese population.

### Haplotype dataset surveys to estimate variant age

The age at which the c.1617del variant arose was estimated using GEVA software (Github commit tag edeafb7) [22] and the haplotype dataset of 3552 Japanese individuals, which consisted of 7104 phased haplotypes and 1,442,809 variants on chromosome 14 [23]. Because GEVA is designed to treat SNPs, the variant chr14:57099780TG>T was modified to chr14:57099780T->G. Using this dataset, we estimated the variant age by GEVA under a recombination model [22] with a generation time of 25 years [24], effective population size of 10,000 [25], and mutation rate of 10^–10^ per site per generation [26, 27].

### Analysis of CHD and renal function in c.1617del heterozygous population

Considering the possibility that not only c.1617del homozygotes, but also heterozygotes may exhibit minor renal phenotypes, a population with the c.1617del variant was selected from ToMMo 54KJPN and compared with the wild-type population for blood data associated with renal function (serum creatinine, serum blood urea nitrogen [BUN], serum uric acid, and serum cystatin C) and for the presence of CHD. Linear regression analysis was performed for creatinine, BUN, uric acid, and cystatin C levels, using sex and age as covariates.

## Results

**Fig. 1:**
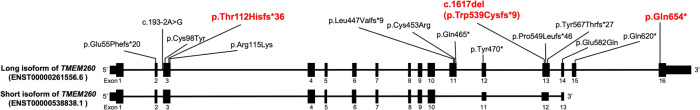
Schematic of the gene structure of long isoform of *TMEM260* (ENST00000261556.6) and short isoform of *TMEM260* (ENST000005338838.1). The variants reported in this study (shown in bold and red) and the pathogenic variants have been published previously [8–12]

The clinical characteristics of each patient with TA from each of the 11 families included in this study are displayed in Table 1. There were five males and six females, with ages ranging from 8 days to 29 years. None of the patients were related to each other, and none were born to a consanguineous couple. Intracardiac repair was completed in all but two infant patients (Patients 1 and 7). We identified putative biallelic pathogenic variants in *TMEM260* in five patients, a de novo heterozygous pathogenic variant in *GATA6* in one patient, and a de novo heterozygous probably pathogenic variant in *NOTCH1* in one patient (Fig. 1, Table 1, Supplementary Table 1). Patients 1−3 harbored the biallelic c.1617del (p.Trp539Cysfs*9) variant in *TMEM260*. A compound heterozygous patient containing a c.1617del variant reportedly exhibited a SHDRA-like phenotype [10]. Patients 4 and 5 had other heterozygous pathogenic variants, c.332dup, p.(Thr112Hisfs*36) and c.1960C>T, p.(Gln654*), respectively, in addition to the heterozygous c.1617del variant. The p.(Gln654*) variant identified in Patient 5 was located in the last exon. This variant is expected to escape NMD but is expected to cause a loss of the end of the TPR domain, which is likely involved in substrate recognition and selective recruitment of IPT domains for O-linked mannose glycosylation (Supplementary Fig. 1) [8]. Additionally, the substantial loss of protein length can affect the stability of the protein. Therefore, we considered p.(Gln654*) to be a likely pathogenic variant. A de novo missense variant, c.1367G>A, p.(Arg456His), in *GATA6* was identified in Patient 6. There have been reports of CHD patients with the same variant [28], and patients with TA or tetralogy of Fallot harboring p.(Arg456Cys) [28]. A de novo missense variant, c.545G>A, p.(Cys182Tyr), in *NOTCH1* was identified in Patient 7. This variant has been reported in a large nonsyndromic tetralogy of Fallot cohort [29] although its pathogenicity has not been functionally evaluated. No additional potential pathogenic variants were identified in this study.

Among the five patients who had *TMEM260* variants, regarding the types of TA, three were type I (Collett and Edwards classification) and type A1 (Vanpraagh classification), and two were type II and type A2 (Table 1). Other cardiac malformations included cor triatriatum and coronary artery fistula (CAF) in Patient 1, right aortic arch (RAA) in Patient 2, persistent left superior vena cava (PLSVC) in Patients 1 and 5, and partial anomalous pulmonary venous return (PAPVR) in Patients 1, 3, and 5. With regard to renal function, no chronic elevation of creatinine levels was observed in Patients 1 through 5. However, Patient 1 developed heart and renal failure after the palliative Rastelli procedure, requiring the induction of dialysis, was unable to withdraw from dialysis, and died. Patients 2–5 have not had renal dysfunction to date, although Patient 5 had hydronephrosis. Neurodevelopmental delay was present in Patients 2 and 4, although in Patient 2 it was unclear whether the delay was congenital or due to posthemorrhagic hydrocephalus. Hearing impairment was observed in Patient 2.

Among the six patients without pathogenic variants in *TMEM260*, two had TA type I and type A1, one had TA type II and type A2, two had TA type A3, and one had TA type III. There were no cases of renal dysfunction. Neurodevelopmental disorders were observed in three patients: Patient 6 had dysplasia of the cortical gyrus and language developmental delay, Patient 8 had post hemorrhagic hydrocephalus, and Patient 9 had hyperactive tendencies. Hearing impairment was observed in Patient 11. Patient 6 had no symptoms suggestive of pancreatic abnormalities, a common extracardiac feature of *GATA6*-related disorders [30].

**Fig. 2:**
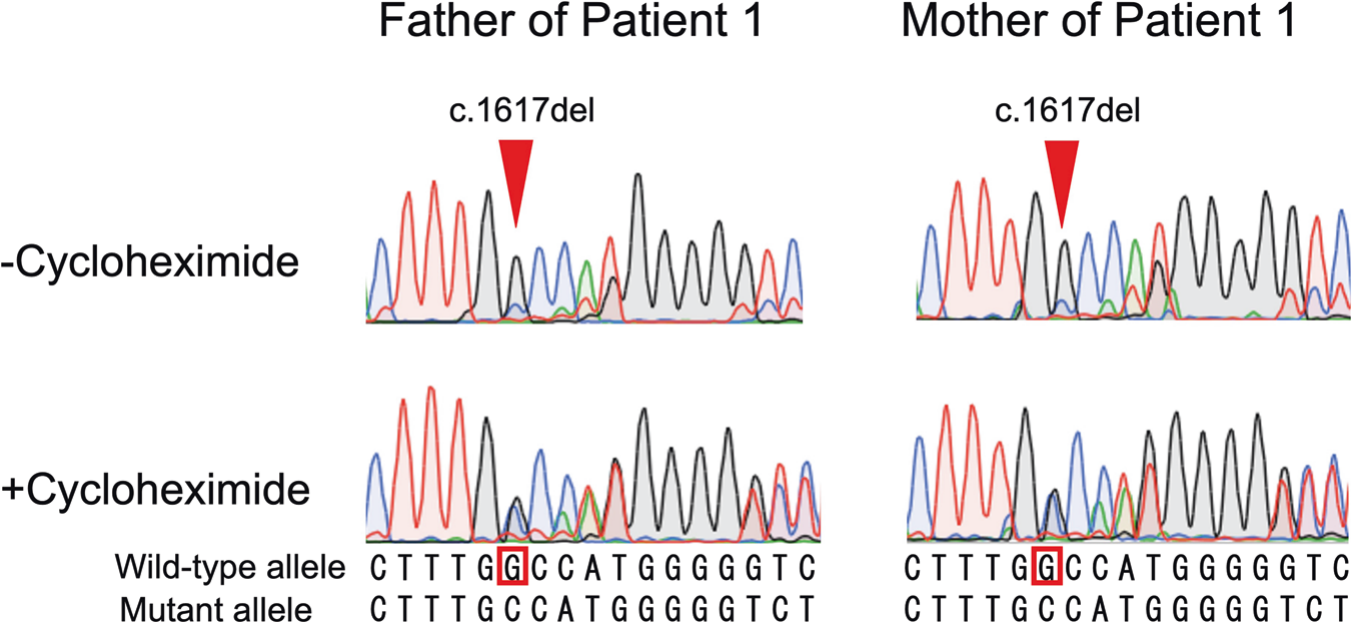
The frameshift variant c.1617del in *TMEM260* leads to NMD. EBV-LCLs from the parents of Patient 1 were treated with cycloheximide to suppress NMD. Sanger sequencing of the c.1617del variant was also performed. The red arrowheads indicate the location of c.1617del. The upper and lower panels show the results before and after CHX loading, respectively

We inhibited NMD using cycloheximide to test whether the c.1617del variant leads to NMD. The c.1617del (p.Trp539Cysfs*9) transcript was detected at much lower levels than the wild-type alleles in EBV-LCLs from the parents of Patient 1. Suppression of NMD by cycloheximide treatment increased the levels of frameshift transcripts. These results indicate that frameshift alleles led to unstable transcripts that were subject to NMD (Fig. 2).

In the Japanese population, the allele frequency of c.1617del was much higher (0.36%) than that in other populations (Table 2). Although less frequent than in the Japanese population, the allele frequency in the South Korean population was also elevated (0.14%; Table 2). In contrast, the variant was absent in the databases we analyzed for the Chinese, Taiwanese, Thai, and Vietnamese populations.

**Table 2:** Allele frequency of the c.1617del variant and estimated incidence of homozygous c.1617del variant in the Japanese and other populations

Population	Japanese	African/African American	Latino/Admixed American	East Asian	Non-Finnish European	Chinese	Taiwanese	South Korean	Thai	Vietnamese
Public genomic databases	ToMMo 54KJPN	gnomAD v2.1.1	gnomAD v2.1.1	gnomAD v2.1.1	gnomAD v2.1.1	The Chinamap	Taiwan Biobank (TWB1)	KOVA2	T-Rex	1KBG
Number of samples	54,302	12,467	17,703	9974	64,517	10,558	27,735	5305	1092	1008
Allele frequency	0.00361	-	-	0.000301	-	-	-	0.00142	-	-
Estimated incidence	1:76,700	-	-	1:11,000,000	-	-	-	1:496,000	-	-

We estimated the age of the c.1617del variant. The allele frequency in the haplotype dataset of 3,552 Japanese was 0.32% (23/7104 haploids). The genotype frequencies were 99.4% for the reference homozygous genotype and 0.6% for the heterozygous genotypes. No alternative homozygous individuals were identified in the dataset. The c.1617del variant occurred approximately 23 kiloyears ago (kya). This estimate did not change when the mutation rate was changed by a factor of 100 or 0.01 (22 kya for both 10^–8^ and 10^–12^). The estimate was also not significantly affected when the generation time was changed to 20 or 30 years (18 kya and 27 kya, respectively). These results suggest that the variant occurred at approximately 23 kya.

Among the 54,302 Japanese individuals participating in ToMMo 54KJPN, the data on serum creatinine, BUN, and uric acid were available for 38,207 individuals, and serum cystatin C was available for 38,206 individuals. Of these, 274 had the c.1617del heterozygous variants. There were no significant differences between individuals with heterozygous c.1617del variants and those with the wild-type allele in any of the above indices (Supplementary Fig. 2). Data on the presence of CHD were available for 13,508 individuals, of whom 88 were heterozygous for the c.1617del variant. None of the heterozygous c.1617del individuals had CHD. None of the individuals in the ToMMo 54KJPN cohort were homozygous for the c.1617del variant.

## Discussion

In this study, we identified a prevalent loss-of-function variant of *TMEM260*, c.1617del, in 8/22 alleles from the 11 Japanese TA patients analyzed who did not have a 22q11.2 deletion: three patients were homozygous for c.1617del variants and two patients harbored compound heterozygous variants of *TMEM260*. Among our patient cohort, we also identified one de novo *GATA6* variant and one de novo *NOTCH1* variant. Based on the allele frequency of the c.1617del variant (0.36%) in the Japanese population, the incidence of biallelic c.1617del variants in the Japanese population was calculated as 1 in approximately 76,700 births (Table 2). Collectively, approximately 26% of Japanese patients with TA could be explained by the homozygous c.1617del variants in *TMEM260*, as the incidence of TA in Japan is estimated to be approximately 1/20,000 births, according to a survey of cardiac disease in childhood conducted by the Japanese Society of Pediatric Cardiology and Cardiac Surgery (https://jspccs.jp/wp-content/uploads/rare_disease_surveillance_2020.pdf). Considering the other variants in *TMEM260*, *TMEM260* mutations would account for the largest- or the second largest- portion of TA cases after 22q.11.2 deletion syndrome, in the Japanese population. Our study suggests that a genetic cause, including 22q11.2 deletion and mutations in *TMEM260*, *GATA6*, or *NOTCH1*, can be identified in more than half of Japanese patients with TA, although the exact proportion is unclear due to our exclusion of patients with 22q11.2 deletion from this study.

Previous reports suggested that CHD, particularly TA, is the most consistent phenotype of SHDRA [9, 11]. In terms of the types of TA, patients with *TMEM260* variants in our cohort had type I (Collett and Edwards classification), type A1 (Vanpraagh classification), type II, and type A2, as previously reported. The presence of cor triatriatum and CAF and the higher rate of complicated cases of PAPVR (3/5 patients) and PLSVC (2/5 patients) than previously reported might be new features of patients carrying the c.1617del variant (Table 3).

**Table 3:** Comparison of clinical characteristics of patients with *TMEM260* variants in this study and those previously reported

	Patients 1−5 in this study	Ta-Shma et al. [9]	Pagamenta et al. [11]	Kuroda et al. [10]	Larsen et al. [8]	Peng et al. [12]
Number of patients	*n* = 5	*n* = 4	*n* = 8	*n* = 1	*n* = 2	*n* = 1
Race	Japanese	Ashkenazi Jewish, Arabic	White British, Pakistani, Ashkenazi Jewish, Sudanese, Chinese	East-Asian	Native American, Pakistani	Chinese
Gender	2 males, 3 female	2 males, 2 females	5 males, 3 females	a male	2 males	1 female
Age	3 months to 29 years	6 weeks to 2 years	3 months to 5 years	10 years	5 years, 7.5 years	8 months
Number of tested families	2 solo, 1 duo, 2 trio	1 duo, 1 sextet	2 trio, 1 quartet, 1 quintet, 1 sextet	1 trio	1 trio, 1quartet	1trio
Inheritance	3 inherited, 2 not provided	inherited	inherited	inherited	inherited	inherited
Deceased	1/5	3/4	6/8 (3 patients had been terminated during pregnancy)	0/1	0/2	0/1
TA	5/5	2/4	8/8	1/1	2/2	1/1
Type of TA (Collett and Edwards classification)						
Type I	3/5	1/2	7/8			1/1
Type II	2/5				1/2	
not reported		1/2	1/8	1/1	1/2	
Combined cardiovascular malformation with TA						
Cor triatriatum	1/5	0/4	0/8	0/1	not reported	0/1
CAF	1/5	0/4	0/8	0/1	not reported	0/1
RAA	1/5	1/4	1/7	0/1	not reported	1/1
PAPVR	3/5	1/4	1/8	0/1	not reported	0/1
PLSVC	2/5	1/4	0/8	0/1	not reported	0/1
Neurodevelopmental delay	2/4 (posthemorrhagic hydrocephalus, autism spectrum)	not reported	1/3	1/1	2/2	1/1
Renal failure	1/5	not reported	3/5	0/1	0/2	0/1
Anuria/Oligria	1/5	2/4	1/4	0/1	0/2	0/1
Elevated creatinine level (normal:0.2−0.9 mg/dl)	0/5	3/3	3/5	0/1	not reported	not reported
Proteinuria	0/5	not reported	0/3	not reported	not reported	not reported

*TA* Truncus arteriosus, *CAF* Coronary artery fistula, *RAA* Right aortic arch, *PAPVR* Partial anomalous pulmonary venous return, *PLSVC* Persistent left superior vena cava

This study suggests that the phenotype and severity of patients with *TMEM260* variants may be more variable than previously reported [9, 11]. Most previous reports have shown a high rate of renal dysfunction and death at a young age in patients with SHDRA [9, 11], although recent reports have described several SHDRA patients with relatively mild symptoms [8, 10, 12]. In contrast, among the patients harboring TMEM260 variants in this study, four of the five survived from 6 to 29 years of age without any signs of renal dysfunction. In particular, Patient 3, at 29 years of age, is the oldest patient with *TMEM260* variants reported to date (Table 3) and has not only had no renal dysfunction but also has no noted developmental delays, graduated from university, and works as a caregiver. Therefore, these results suggest that some patients with *TMEM260*-related CHD can survive long-term without obvious developmental or renal dysfunction.

The c.1617del variant is estimated to have occurred at 23 kya. The divergence between European and Asian populations is estimated to be 20–40 kya [27], whereas the divergence between Han Chinese and Japanese populations is estimated to be 8–9 kya [27]. Thus, the estimate of 23 kya is broadly consistent with the notion that the variant occurred around the time the Asian population arose and was enriched in the South Korean and Japanese populations by chance, especially in the Japanese population. One limitation of our estimation method was the utilization of the GEVA software for indel variants. However, the fact that changing the mutation rate in the 10,000-fold range resulted in nearly identical estimates suggests that the estimates did not deviate significantly from the true variant age.

Our analysis of the c.1617del heterogeneous population showed no significant differences in blood test values for renal function compared with the wild-type population, and none of the individuals had CHD. To the best of our knowledge, no studies have analyzed large *TMEM260* variant carrier populations. Further comprehensive analyses are required to evaluate the effects of heterozygous *TMEM260* variants.

In conclusion, we identified a prevalent loss-of-function variant, c.1617del in *TMEM260*, among Japanese TA patients. Genetic testing for *TMEM260*, including the c.1617del variant, should be considered in Japanese TA patients lacking 22q11.2 deletion. Simultaneously, researchers at Keio University independently reported the same c.1617del variant of *TMEM260* as a major genetic cause of TA in Japan, resulting in the same conclusion as that of our study (Prof. H. Yamagishi at Keio University and A. Kikuchi, personal communication). We propose to name the c.1617del variant as “Keio-Tohoku variant of *TMEM260*” considering its importance for the Japanese population.

### Supplementary information

Supplementary Figures

Supplementary table 1

ICMJE Disclosure Forms
