# Correction: Genetic etiology of truncus arteriosus excluding 22q11.2 deletion syndrome and identification of c.1617del, a prevalent variant in *TMEM260*, in the Japanese population

**DOI:** 10.1038/s10038-024-01245-6

**Published:** 2024-03-29

**Authors:** Hisao Yaoita, Eiichiro Kawai, Jun Takayama, Shinya Iwasawa, Naoya Saijo, Masayuki Abiko, Kouta Suzuki, Masato Kimura, Akira Ozawa, Gen Tamiya, Shigeo Kure, Atsuo Kikuchi

**Affiliations:** 1 Department of Pediatrics, Tohoku University Graduate School of Medicine, Sendai, Japan; 2 Department of Pediatric Cardiology, Miyagi Children’s Hospital, Sendai, Japan; 3 Department of AI and Innovative Medicine, Tohoku University Graduate School of Medicine, Sendai, Japan; 4 Tohoku Medical Megabank organization, Tohoku University, Sendai, Japan; 5 Statistical Genetics Team, RIKEN Center for Advanced Intelligence Project, Tokyo, Japan; 6 Department of Rare Disease Genomics, Tohoku University Graduate School of Medicine, Sendai, Japan; 7 Department of Pediatrics, Yamagata University Graduate School of Medicine, Yamagata, Japan; 8 Miyagi Children’s Hospital, Sendai, Japan

**Keywords:** Congenital heart defects, Haplotypes, Clinical genetics, Next-generation sequencing, Medical genomics

Correction to: *Journal of Human Genetics* 10.1038/s10038-024-01223-y, published online 13 February 2024

The original article stated in its Ethics Declarations, “Some of the authors declared Financial and Non-Financial Relationships and Activities, and Conflicts of Interest regarding this manuscript as indicated in the supplementary materials.” However, due to an error on the part of the publisher, the Conflicts of Interest were not included in the supplementary materials for this article. The ICMJE Conflict of Interest Disclosure Forms from the authors of the article have been added to the electronic supplementary materials.

The following funding statement has also been added to the article: “This research was supported in part by the Japan Agency for Medical Research and Development, AMED under Grant Number JP21tm0124005 and JP23tm0424402. Drs. Takayama, Tamiya, Kure, and Kikuchi have received a research grant from Astellas Pharma.”

The original article has been corrected.

### Supplementary information

ICMJE Disclosure Forms

