# Supplementary material for: Genetic etiology of truncus arteriosus excluding 22q11.2 deletion syndrome and identification of c.1617del, a prevalent variant in TMEM260, in the Japanese population
Source: J Hum Genet. 2024 Feb 13;69(5):177–83. doi: 10.1038/s10038-024-01223-y (PMC11043042; doi:10.1038/s10038-024-01223-y)
Supplement: Supplementary file 1 — Supplementary Figures [file 10038_2024_1223_MOESM1_ESM.pdf]

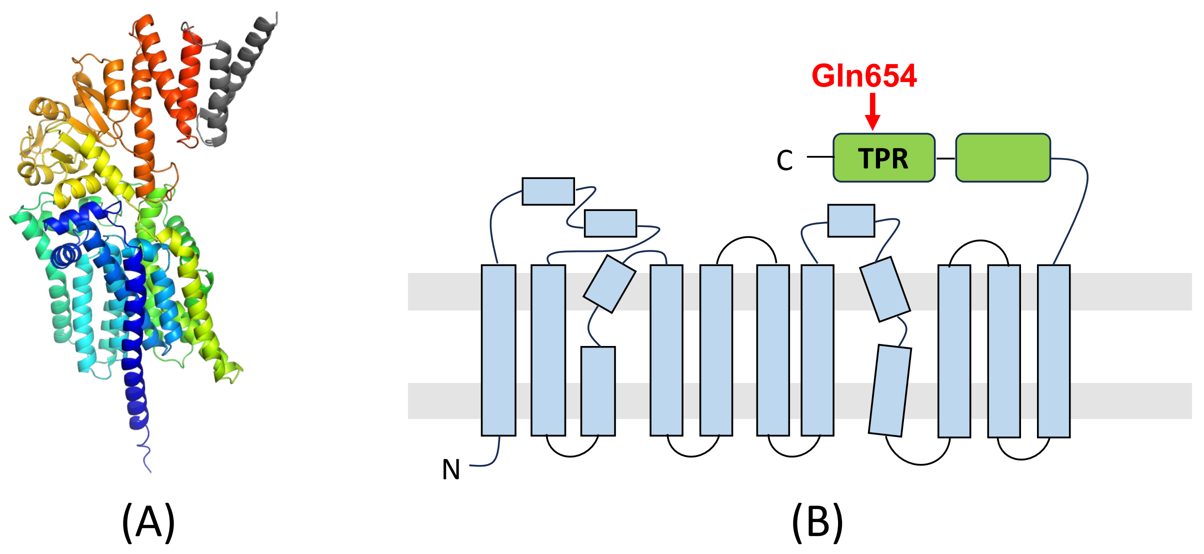

Supplementary Fig. 1 Location of TPR domain and p.Gln654* in the TMEM260

(A) Three-dimensional structure of human TMEM260 protein. The molecular model is based on data predicted by AlphaFold2 (AF-Q9NX78-F1-model_v4) [1, 2] and visualized using PyMOL 2.5.7. Residues that predicted to be lost due to p.Gln654* were colored in gray.

(B) Schematic diagram of the location of p.Gln654 in TMEM260 protein. The location of the domains is based on a previous report.[3]

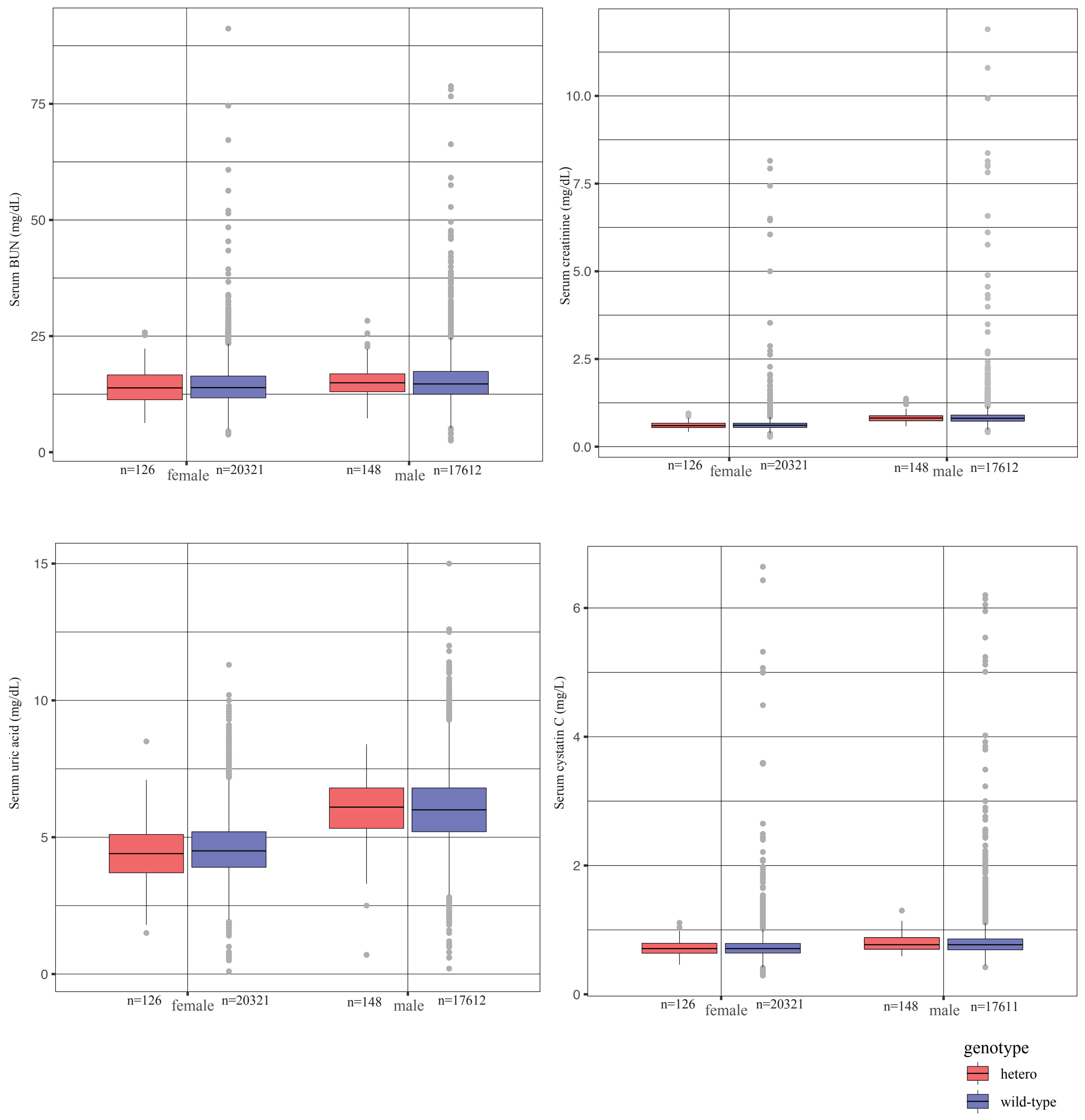

Supplementary Fig. 2
Box plot of c.1617del heterozygous and wild-type populations about serum BUN (P = 0.925), serum creatinine (P = 0.744), serum uric acid (P = 0.252) and serum cystatin C (P = 0.947). Horizonal line represents median, box interquartile range, and whiskers show 1.5× the interquartile interval.

Supplementary Reference

1. Jumper J, Evans R, Pritzel A, Green T, Figurnov M, Ronneberger O, et al. Highly accurate protein structure prediction with AlphaFold. Nature. 2021;596:583–9.

2. Varadi M, Anyango S, Deshpande M, Nair S, Natassia C, Yordanova G, et al. AlphaFold Protein Structure Database: massively expanding the structural coverage of protein-sequence space with high-accuracy models. Nucleic Acids Res. 2021;50:D439–44.

3. Larsen ISB, Povolo L, Zhou L, Tian W, Mygind KJ, Hintze J, et al. The SHDRA syndrome-associated gene TMEM260 encodes a protein-specific O-mannosyltransferase. Proc Natl Acad Sci U S A. 2023;120:e2302584120.
